# The Impact of Social Support and Pregnancy on Subjective Well-Being: A Systematic Review

**DOI:** 10.3389/fpsyg.2021.710858

**Published:** 2021-09-09

**Authors:** Buyantungalag Battulga, Marc Reginald Benjamin, Hong Chen, Enkhmandakh Bat-Enkh

**Affiliations:** ^1^Department of Psychology, Southwest University, Chongqing, China; ^2^Department of Agricultural and Applied Economics, Mongolian University of Life Science, Ulaanbaatar, Mongolia; ^3^School of Politics and Public Administration, Southwest University, Chongqing, China

**Keywords:** systematic review, subjective well-being, social support, pregnancy, quality of life

## Abstract

**Background:** Subjective well-being (SWB) has a protective role in mental health maintenance and is prone to change during short stressful moments, such as pregnancy. Longstanding research suggests that social support (SS) from the partner and family members of pregnant women directly or indirectly acts as a buffer against negative mental outcomes. For happier pregnancies, it is important to understand how SS and pregnancy affect the SWB.

**Objective:** This review aims to examine the extended association of being pregnant and SS on the SWB of pregnant women.

**Methods:** A systematic review was conducted in PubMed, ScienceDirect, and Google Scholar. Articles published in peer-reviewed journals were included regardless of the year and if they had assessed the impact of at least one SWB or SS outcome among healthy pregnant women. The tools of the National Heart, Lung, and Blood Institute were used for quality assessment.

**Results:** Thirty-four studies that assessed the domains of SWB measurements, such as happiness, quality of life (QoL), life satisfaction, positive and negative effects, and well-being, were included and its association with either pregnancy or SS was summarized. Variable results, such as life satisfaction, happiness, and mental component of QoL, were found to be high during pregnancy, but positive emotion and physical components of QoL had decreased. Almost universally, SS during pregnancy was found to have a positive association with all measurements of SWB.

**Conclusion:** This study had found that, despite some arising trends, pregnancy itself does not necessarily have similar impacts on SWB across healthy pregnant women. However, SS had a significant effect on SWB.

## Introduction

Within multiple studies in the literature, research has yielded an association between subjective well-being (SWB) and social support (SS) with mental health (Umberson and Montez, 2010; Steptoe et al., 2015; Ngamaba, 2017; Tough et al., 2017; McDonald, 2018). There is extensive evidence about the importance of mental health during pregnancy. The majority of these studies focused on mental health conditions such as depression and anxiety (Psaros et al., 2009; O'Connor et al., 2016; Zegeye et al., 2018; Yasuma et al., 2019; Ponting et al., 2020). However, it is important to consider positive psychology and its positive effect on mental health and well-being within pregnant women (Park et al., 2014; Giangiordano et al., 2020). It has been hypothesized that social relationships directly protect mental health or indirectly act as a buffer against stressful circumstances (House et al., 1988). Furthermore, the importance of strong social ties for life satisfaction and SWB caused the WHO to identify SS as a key determinant of active aging (WHO, 2002).

Subjective views of people of their life experiences and perceptions of existence (including effective reactions and cognitive judgments) are referred to as SWB (Diener, 1984; Russell, 2008; Martín-María et al., 2017; Jebb et al., 2020). The three different aspects of SWB are evaluative, hedonic, and eudemonic well-being (Steptoe et al., 2015). The evaluation of how content people are with their lives, such as life satisfaction (LS) and work satisfaction, is referred to as evaluative well-being. Feelings or moods such as happiness or pleasant effects are indicated under hedonic well-being. Eudemonic well-being is about the judgment on the meaning and purpose of life (Steptoe et al., 2015). The terms QoL, happiness, and LS are used interchangeably (Ngamaba et al., 2017), and there is heterogeneity in measurements and concepts used in the field of positive psychology. As from the perspective of the individual and community experiencing well-being, many of those measurements may overlap with each other, but not fully; each one captures, as a measured variable, distinct aspects of SWB. The inclusion or exclusion of the measurement QoL to SWB is in many studies mentioned as a prominent aspect of SWB (Diener et al., 2006; Camfield and Skevington, 2008; Steel et al., 2008; Medvedev and Landhuis, 2018), although it is not included in all of them (Steptoe et al., 2015). As many scholars find the mental component of QoL an important aspect of SWB, some researchers maintain that it is as important as the three aspects of SWB (Sakuraya et al., 2020).

Although there are several studies that focus on a particular aspect of SWB in physical and mental health (Zautra and Hempel, 1984; Vothknecht et al., 2011; Ngamaba et al., 2017; Mansfield et al., 2018; Vescovelli et al., 2018; Buecker et al., 2020; Moura and Hamdan, 2020; Tilley et al., 2020; Todd et al., 2021), studies on pregnancy and pregnant women have received little attention. Several reviews have looked at the quality of life (QoL) of pregnant women (Mogos et al., 2013; Lagadec et al., 2018), yet none have specifically focused on the association between pregnant women and SWB. Furthermore, in previous meta-analyses and reviews, perinatal maternal or postpartum depression is much more widely researched (Alder et al., 2007; Grigoriadis et al., 2013; Biaggi et al., 2016; Seth et al., 2016; Gentile, 2017). Previous studies assessed the factors influencing the QoL of pregnant women (Lagadec et al., 2018) and the quality of tools to measure QoL among pregnant and postpartum populations (Mogos et al., 2013). They discovered that while the physical aspect of QOL declined, the mental aspect remained steady and improved throughout pregnancy (Lagadec et al., 2018). An increased QoL was related to a lack of social and economic issues, having family and friends, feeling happy about being pregnant, and being optimistic (Lagadec et al., 2018). Due to the broad definition of SWB, studies being assessed under the same perspective of well-being might not be found under the same keyword. Hence, this review included all aspects of SWB, which includes happiness, QoL, LS, and positive and negative effects. For global mental health, the improvement of SWB is one of the major concerns (Steptoe et al., 2015). Studies indicate that higher levels of SWB are associated with more adaptive dispositions and temperaments, more functional health statuses, fewer symptoms of mental illness, stronger interpersonal relations, more self-enhancing cognitive styles, and more prosocial functions (Diener, 1984; Diener et al., 1995; Lyubomirsky et al., 2005; Pressman and Cohen, 2005). At the time of this writing, maternity care policies emphasize the need for fostering emotional well-being along with physical health (Jomeen, 2004).

Since the early 1970s, the term SS has been appraised in various studies of health and well-being (Tsouna-Hadjis et al., 2000) and is a complex, multifaceted construct (Uchino, 2004). According to the APA Dictionary of Psychology, it is defined as “the provision of assistance or comfort to others, typically to help them cope with biological, psychological, and social stressors” (APA, 2020). SS can be described through two different concepts, namely, perceived or received (Cobb, 1976) and structural or functional (Shumaker and Brownell, 1984). Perceived SS relates to perceptions of the general availability of support (Schwarzer et al., 2004; Haber et al., 2007), as both satisfaction with the support and the availability of it (Sarason et al., 1990). Received SS, on the other hand, relates to the actual recognized instance or measure of received supportive behavior of an individual (Haber et al., 2007). Overall, it is possible to consider perceived and received support as theoretically separate and marginally related (Schwarzer et al., 2004; Haber et al., 2007). The quantity and types of connections inside the social network of an individual are referred to as structural SS (e.g., size of the social network, network composition, and frequency of contact with network members) (Uchino et al., 1996; Ford et al., 1998; DiMatteo, 2004; Haber et al., 2007). Functional SS, on the other hand, is defined as the exchange of emotional (e.g., encouragement), instrumental (e.g., housekeeping), or informational (e.g., notifying someone of a job opportunity) aid received from others (House, 1981; Thoits, 1982; House et al., 1988). SS is concerned with the function and quality of social relationships in general (Schwarzer et al., 2004). By contrast, there are various definitions of loneliness. One definition in particular defines loneliness as “a distressing feeling that accompanies the perception that one's social needs are not being met by the quantity or especially the quality of one's social relationships” (Weeks, 1994; Hawkley et al., 2008; Yu et al., 2020). A portion of the literature discovered that isolation and aloneness are synonymous with loneliness (Banet, 1978; Peplau and Perlman, 1982); however, according to studies, people must view themselves as lonely to experience loneliness, regardless of their physical or social surroundings (West et al., 1986; Liu et al., 2016). Simultaneously, investigations into social network size, social network cohesiveness (density), and loneliness may yield crucial insights, which could pose a tremendous contribution to the literature (Newall and Menec, 2017). Hence, this review focuses on loneliness in the context of SS.

Social support may decrease the risk of depression during pregnancy, and women with more SS had more positive health and better pregnancy outcomes (Wells et al., 1989; Orr et al., 2002; Orr, 2004; Figueiredo et al., 2014). Other than positive pregnancy outcomes, receiving adequate SS has been associated with improvements in her LS and school performance (Stevenson et al., 1999). Due to continued care and support from the partner and the family members of pregnant women, they would be less affected by depression, mental stress, and anxiety disorders (Maharlouei, 2016). Another research had stressed the significance of considering each source of support, whether it comes from parents, peers, spouses, or friends, and its impact on well-being individually (Stevenson et al., 1999). Although it has been broadly accepted that SS has a positive impact on the possible stresses on SWB (Henderson, 1977; Sarason, 1990; Cramm et al., 2010; Khan and Husain, 2010), there are not many publications about pregnant populations. Among the existing publication of SS during pregnancy (Gjerdingen et al., 1991; Orr, 2004; Hodnett et al., 2010; East et al., 2019), many have clearly stated the importance of support on pregnancy and its outcome. However, to our knowledge, the only publication that has assessed both QoL and SS among pregnant women had stated that the satisfaction these women feel of their support has more impact on SWB. Instead of SWB, numerous researches have looked into the association between SS and psychological well-being in pregnant women. The vast majority of these studies were centered on the postpartum period (Thompson, 1986; Colletta, 1987; Kissman and Shapiro, 1990; Thompson and Peebles-Wilkins, 1992; Davis and Rhodes, 1994) or sampled pregnant teens (Barrera, 1981; Sacco and McLeod, 1990; Cynthia Logsdon et al., 2005).

This review included all studies up to spring 2021 that utilized self-reported health and QOL as outcomes, as these are the frequently used metrics of subjective health. The research findings comprised within this paper are a systematic review of research on SS and pregnancy in SWB published between 1985 and 2021. It focuses on studies that look at differences in self-rated SWB, happiness, QoL, life satisfaction, and loneliness. Hence, this review expands on the much-needed SWB literature and pregnant women, as well as about the impact SS has on pregnancy.

## Methodology

**Figure 1:**
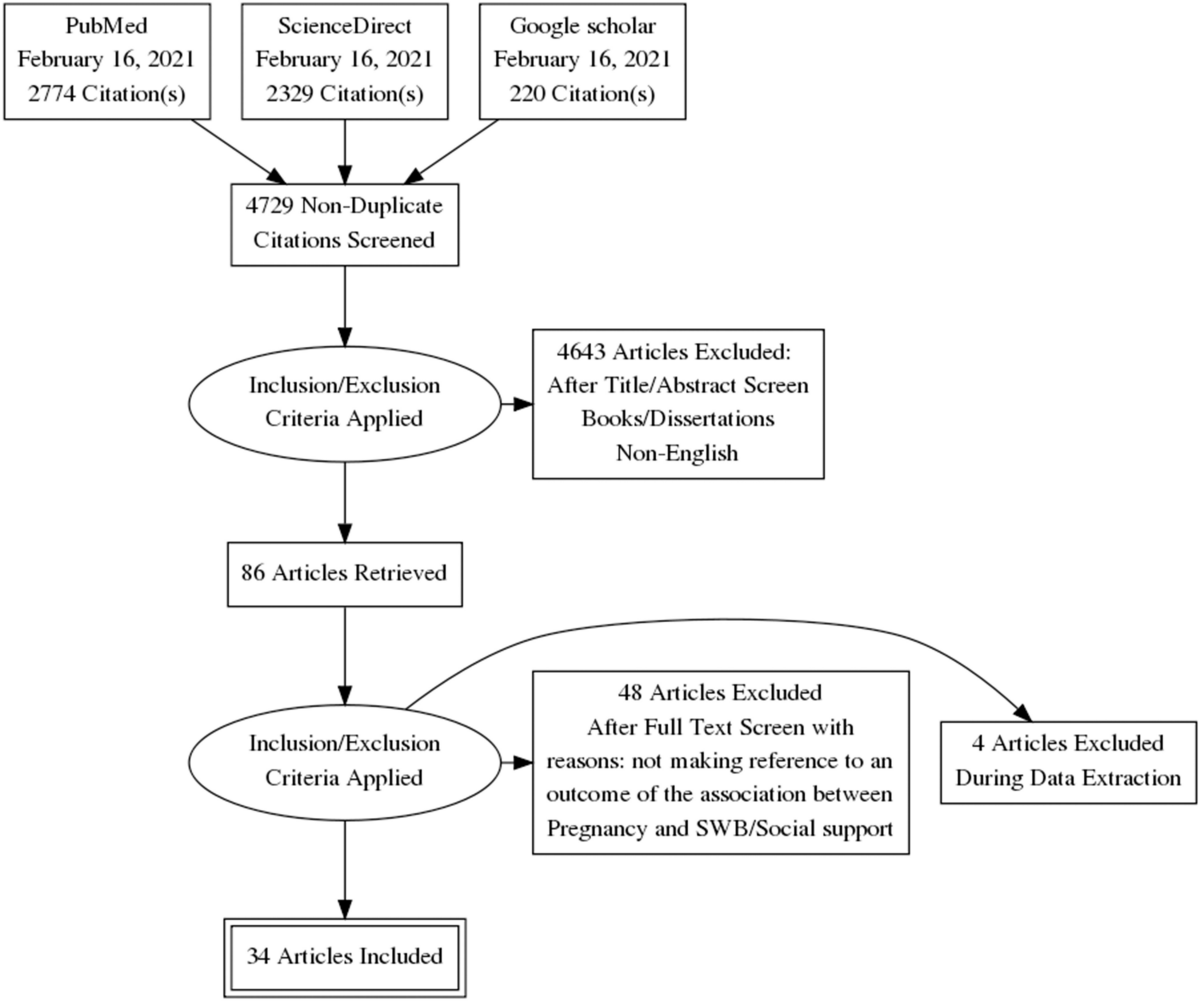
Flowchart of the systematic literature search.

The search strategy and search terms were based on the participants, intervention, comparison, outcomes, and study design (Higgins et al., 2019). The preferred reporting items for the systematic reviews and metaanalyses checklist were used to complete this review (Tricco et al., 2018; Figure 1).

### Inclusion and Exclusion Criteria

Predefined inclusion criteria included articles with the following key factors: (i) Population: healthy pregnant women, without previous mental or physical complications, except for transitions into disease; (ii) intervention (or exposure to observational studies): pregnancy itself is at the center of these studies, with at least one measurement of any form of SWB; the health, education, and natal health promotion interventions that led to improvements in the SWB of the target; (iii) comparison: particularly of nonpregnant female participants and participants who did not receive health education and health promotion interventions; (iv) outcome: changes in any form of SWB; (v) studies: observational studies (e.g., cohort, cross sectional, case-control) as well as qualitative studies (interviews); and (vi) published in English; (vii) published prior to February 2021 with no restrictions in the starting date, regardless of ethical group or geographical origin.

Nonempirical articles (such as abstracts, conference submissions, and editorials), dissertations, and thesis papers were omitted.

### Search Strategy and Data Sources

Systematic, yet broad, searches of the literature were carried out using the following electronic databases: PubMed, Google Scholar, and ScienceDirect. Search terms were derived from the theoretical consideration of the writer, as well as terms in accordance with the previous review. The following main key terms were used: pregnancy and SWB, happiness, life satisfaction, well-being, and QoL. The search strategy is displayed in Table 1. Results were obtained from the initial search from Set 4. The search was limited to title or abstract, as without it, the search became too broad. For each database, the search strategy was changed to generate the desired papers.

**Table 1:** Search term for the literature search.

**Set**	**Search terms**
1	“social network” OR “social support” OR “social connection” OR “social isolation” OR “loneliness”
2	(“happiness” OR “Quality of life” OR “subjective well-being” OR “life satisfaction)”
3	“pregnant” OR “pregnancy”
4	[((“social network” OR “social support” OR “social connection” OR “social isolation” OR “loneliness”) AND “pregnant”) OR “pregnancy”] AND (“happiness” OR “Quality of life” OR “subjective well-being” OR “life satisfaction”)

### Study Selection

A database search was conducted on February 16, 2021. The preliminary findings were stored in the endnote citation manager and integrated into one file. Duplicates and foreign articles were removed. Two independent researchers screened the title and abstract of the identified papers for their relevance. The complete texts of papers deemed “relevant” for the review were then obtained and compared with our inclusion criteria. The full-text screening was performed by one researcher, and the results were discussed. Until a census was obtained, any differences were carefully explored in the group meetings.

### Data Extraction and Synthesis

For data extraction, an excel file was created. The following features were extracted: (i) name of the first author and year of publication; (ii) the country in which the research was carried out; (iii) research design; (iv) sample size and participant characteristics; (v) the type of assessment instrument (SWB, happiness, life satisfaction, and life quality); and (vi) results and conclusions. We focused on the data relevant to our research topic if articles addressed several research issues. Summaries of the characteristics and results of each outcome of the included studies were organized in a tabular form (Tables 2, 3).

**Table 2:** The effect of pregnancy on subjective well-being (SWB).

	**Author (Year) and Design**	**Country/setting**	**Sample size**	**Mean age**	**Pregnancy state**	**Covariant**	**Dependent variable/SWB scale**	**Main result on SWB outcome**
(1)	Taşdemir et al. (2010) and Cross sectional	Turkey/Primary health centers	147 adolescents, 156 adults	18.0 ± 0.1, 23.9 ± 0.3	Pregnant adolescents (<20 years of age), pregnant adults (20–29 years of age)	Age, age at first marriage, multiparity, gestational period, consanguineous marriage, social insurance, smocking, education, and economic status	QoL/SF-36	All QoL scores, except the bodily pain scale, were lower among the adolescents when compared to the adults. The QoL scores were highest in the second trimester for both groups.
(2)	Robinson et al. (2018) and Cross sectional	Norway/Maternity care units	283	31.3 ± 4.2	Gestation week 15, 30, and at 12 weeks and 1 year postpartum	Age, weight, height, BMI, education, pain intensity, disability, marriage, employment, and parity	HRQoL/SF-36, NHP questionnaire	Statistical significant differences in all domains of both HRQoL instruments in late pregnancy compared to the expected age-adjusted means of the reference populations except for Social isolation
(3)	Drescher et al. (2003) and Cross sectional	Texas/Prenatal care at the University Hospital	42	16	Pregnant teens	Age, education, live with family, parity, and population	QoL/MOS-SF 36v2	Pregnant adolescent girls had slightly lower overall scores for all components of perceived QoL, except the vitality component. Scores were significantly lower than normative scores for only the physical functioning component.
(4)	Chang et al. (2014) and Cross sectional	Taiwan/ Medical center	358	N\A	≤ 16 weeks of gestation	Employment, marital status, education, gravidity, parity, abortion, infertility, assisted reproduction, medical condition, expected pregnancy, and income (personal, household)	HRQoL (MCS)/SF-36	The significant factors predicting the MCS scores during pregnancy included stage of pregnancy, parity, and medical condition. The factor predicting the mental health scores was a medical condition.
(5)	Tesfa et al. (2020) and Cross sectional	Ethiopia/General Hospital	1,606	≤ 19 (7.0%), 20–29 (64.5%), 30–39 (25.3%), ≥40 (3.2%)	Pregnancies in the last 6 months, without complications that need admission to a hospital	Residence, age (maternal, partner), age during first pregnancy, education (maternal, partner), occupation (maternal, partner), marital status, and family size	Happiness/OHQ	The prevalence of happiness was 24%. Mothers who were exposed to unintended pregnancy reported intimate partner violence and being in the third trimester were the most predictor variables with a low level of happiness.
(6)	Türk et al. (2017) and Cross sectional	Erzurum/Maternity hospital	272	17–25 (47.0%), 26–34 (39.7%), 35 and over (13.4%)	Pregnancies without exclusion	Age (maternal, partner), education (maternal, partner), marriage age, working status (maternal, partner), income status, living place, and family type	Happiness/OHQ-SF	Getting pregnant for the first time at the age of 26–35, having no stillbirths, and having a planned pregnancy were found to increase the level of happiness.
(7)	Ishfaq and and Mushtaq (2019) and Cross sectional	Pakistan/Gynecology Wards of hospitals, Private clinics	204	27 ± 1.34	Had at least one child and no physical or mental disabilities	Age	Happiness/The Subjective Happiness Scale-Urdu	Younger women have more subjective happiness than elder pregnant women.
(8)	Malhotra et al. (2015) and Cross sectional	India/Nursing homes, dispensaries, child care clinics	100	28.73	Pregnancies without exclusion	Age, family size, family type, working status, trimester, order of pregnancy, and past medical complications	Happiness/OHQ	Pregnancy emerged as a period that elicits an increase in the emotional states of women, with average happiness of 74.01% among Indian women
(9)	Ndombe et al. (2011) and Longitudinal	Norway/based on the Norwegian Mother and Child Cohort Study	67,355	30.0 ± 4.6	Routine ultrasound examination around 18 weeks of gestation	N/A	LS/SWLS, five-item Relationship Satisfaction scale	LA increased with almost 0.1 standard deviations during pregnancy and infancy to reach its highest level at 6 months postpartum. SA changed more across time than satisfaction with the relationship and that satisfaction with life and with the relationship were more highly related as time passed
(10)	Branecka-Wozniak et al. (2020) and Cross sectional	Poland/Pregnancy pathology ward, Independent Public Clinical Hospital	181	29.22 ± 3.88	Different trimesters	Age, place of residence, education, employment status, trimester, first pregnancy, having children (number, age), previous delivery, presence of partner at delivery, Kegel's exercise	LS/ SWLS	The most numerous women were those with a high level of life satisfaction.
(11)	Aasheim et al. (2014) and Longitudinal	Norway/based on the National Norwegian Mother, Child Cohort Study	18,565	25–31 (*n* = 13,107) 32–37, (*n* = 4,827) ≥ 38 (*n* = 631)	Routine ultrasound examination at ~17 weeks of gestation	Age, single status, native language, employment status, income, pregravid BMI, smoking, obstetric and infant outcomes (IVF, mode of delivery, preterm, neonatal transfer)	LS/SWLS	Satisfaction with life decreased from around age 28–40 and beyond. When comparing women of advanced and very advanced age with the reference group, satisfaction with life was slightly reduced in the two older age groups and most of all in women of very advanced age.
(12)	Farooq et al. (2018) and Cross sectional	Pakistan	100 pregnant women, 100 nonpregnant women	N\A	Pregnancies without exclusion	N/A	PAandNA/Positive Affect and Negative Affect Scale, Brief Cope Scale	Active avoidance coping is negatively correlated with positive affect and positively correlated with negative affect. Emotion-focused coping and problem focus coping do not predict positive affect and negative affect across pregnant women.
(13)	Lips (1985) and Longitudinal	Various places (Obstetricians, prenatal classes, parenting classes, church, etc.)	105 pregnant women and 95 of their husbands, 151 nonpregnant women and 116 of their husbands	N\A	Pregnant and nonpregnant women	Age, mean duration of marriage, and education	PAandNA/55 item Symptom Checklist (XL), Beck Depression Inventory	A number of women experience an increase in negative emotionality over the course of pregnancy and into the early postpartum

*QoL, Quality of Life; HRQoL, Health-related Quality of Life; MCS, Mental component summary; LS, Life Satisfaction; PAandNA, Positive Affect and Negative Affect; SF-36, 36-Item Short Form Survey; NHP, Nottingham Health Profile; MOS, Medical Outcomes Study; OHQ, The Oxford Happiness Questionnaire; SWLS, Satisfaction with Life Scale*.

**Table 3:** The effect of social support (SS) on SWB among pregnant women.

	**Author (Year) and Design**	**Country/setting**	**Sample size**	**Mean age**	**Covariable**	**Social support outcome measurement**	**Dependent variable/SWB scale**	**Result on SWB Outcome**
(1)	Emmanuel et al. (2012) and Longitudinal	Australia/Public hospitals	473	18–24 (28.40%), 25–29 (32.4%), 30–45 (38.8%)	Age, length of relationship, partner, education, first antenatal visit, parity, childbirth, education classes	The Maternal Social Support Scale	HRQoL/SF-12	Social support was found to be a significant and consistent predictor of higher HRQoL scores, particularly in the physical domain at 12 weeks following childbirth and mental domain during the perinatal period. The other significant predictor was the length of relationship with the partner in the mental domain at 36 weeks of pregnancy.
(2)	Ngai and Ngu (2013) and Cross sectional	China/Antenatal clinic	267	32.0 ± 4.4	Age, education, employment status, household income, and gestation	Family Sense of Coherence Scale, Social Readjustment Rating Scale	HRQoL/SF-12	Family sense of coherence and social support had a direct impact on the mental health component of QoL and depressive symptoms during pregnancy.
(3)	Gul et al. (2018) and Cross sectional	Pakistan/District Headquarters Hospital	120	26.76 ± 4.11	Age, trimester, education, history of illness	Multidimensional scale for perceived social support	HRQoL/SF-36	There was a significantly positive relationship between social support and health-related QoL among the pregnant subjects.
(4)	Elsenbruch et al. (2007) and Cross sectional	Germany/Private practice	896	29.20 ± 5.02	Week of pregnancy, planned pregnancy, age, partnership status and duration, children, education, employment status, BMI, smoking, and health condition	22-item version of the social support questionnaire	HRQoL/CES-D, SF-12	Pregnant women with low support reported increased depressive symptoms and reduced QoL.
(5)	Calou et al. (2018) and Cross sectional	Brazil/Public prenatal care services, a private unit in Fortaleza	261	28	Age, partnership status, education, employment status, income, trimester, prenatal consultation, parity, previous birth	A questionnaire covering sociodemographic, obstetric, and health-related QoL data	HRQoL/Mother-Generated Index	The predictors that positively influenced the QoL: occupation/self-esteem; parity/relationship with the family; partner support/relationship with the partner; marital status/relationship with the partner and persons with whom the woman lives.
(6)	MoghaddamHosseini et al. (2021) and Cross sectional	Hungary/Antenatal clinics at the Department of Obstetrics, Gynecology of the University of Pécs	477	32.41 ± 5.32	Age, education, employment status, marital status, place of residence, economic hardship, gestational age, parity, wanted pregnancy, history of miscarriage, mode of last birth, and psychological characteristic	Multidimensional Scale of Perceived Social Support	HRQoL/PROMIS-43	Social support was a significant predictor for better HRQoL in depression.
(7)	Macleod and Weaver (2003) and Longitudinal	Maternity units' antenatal clinics	99	19.5 ± 3.26	Age, postal area, employment status	The Social Support Questionnaire, Network size and composition, Satisfaction with available support	Happiness/ Happiness with 6-choice item, Attitudes toward the pregnancy 15-item rating scale	The participants felt well supported by their families, and many appeared to be in stable relationships throughout the antenatal period. Positive changes in attitudes toward the pregnancy and fetus between 20 and 37 weeks were found.
(8)	Pakseresht et al. (2019) and Cross sectional	Iran/Teaching hospital in Rasht	480	<18 (0.4%), 18–25 (21%), 26–35 (56.9%), >35 (21.7%)	Age (maternal, husband, marriage), education (maternal, partner), occupation (maternal, partner), marital satisfaction, living with, income, obstetric characteristic	A questionnaire covering their husband's characteristics and support of family/others	Happiness/OHQ	18.3% of women had low happiness levels, 65.4% moderate level, and 16.3% high level of happiness. Significant association with marital satisfaction, family support
(9)	Stevenson et al. (1999) and Cross sectional	Baltimore County/prenatal teen clinics	67 Black and 43 White	16.7	Ethnic composition, age, parity, pregnancy week, and planned pregnancy	Procidano and Heller's 1983 parents and friends scales, Marital Adjustment Scale	LS, Self-esteem/Rosenberg's Self-esteem scale, 5-item LS scale by Diener	The reciprocal exchange of support between parents and teens was correlated with increased mastery and life satisfaction and decreased depression and anxiety. The reciprocal exchange of support with friends did not correlate with well-being. A high-quality relationship with a significant other was associated with increased self-esteem among pregnant teens dating the father of their child.
(10)	Yu et al. (2020) and Cross sectional	China/Community Healthcare Service centers	290	31.88 ± 3.83	Age, educational degree, family per capita monthly income and employment status	Multidimensional Scale of Perceived Social Support	LS/SWLS, SAS, MSPSS	A higher level of perceived social support was related to a higher level of LS. Perceived social support partly mediated the relationship between anxiety symptoms and LS
(11)	Gebuza et al. (2014) and Longitudinal	Antenatal ward, clinic “K,” the private gynecological practice	199	N\A	Marriage status, education, live with partner, age (maternal, partner), gravidity, parity, financial status, employment status, antenatal class, newborn condition and weight	The Berlin Social Support Scales	LS/SWLS	Significant increase in life satisfaction in the postpartum period. An important correlate of life satisfaction in the third trimester of pregnancy is social support received.
(12)	Giblin et al. (1987) and Cross sectional	Prenatal clinic	57	16.6	Age, socioeconomic status, work/school status, pregnancy outcome	Questionnaires assessed living arrangements, sources of financial support, adolescent female's partner, family, friends to the pregnancy, use of health care services, anticipated assistance with child care	Positive emotion/short-form Coopersmith Self-Esteem Inventory	The pleasure with pregnancy was positively associated with the receipt of assistance from the adolescent's mother, favorable opinions of friends, and satisfaction with living arrangements.
(13)	Nakamura et al. (2018) and Longitudinal	Japan/based on the Japan Environment and Children's Study	3,513	30.5	Age at delivery, type of family, marital status, education, income, and employment	The Family APGAR	Positive emotion/MCS, Kessler-6	The extent of positive emotion was significantly related to health-related QoL and satisfaction with family relationships during pregnancy
(14)	Rokach (2007) and Cross sectional	Canada/urban city	91 pregnant women, 97 women during the first year following childbirth, 208 women from the general population	30.4 ± 10.4	Age, marital status, education,	General Questionnaire	Loneliness/The Loneliness Antecedents Questionnaire	Sources of loneliness were significantly different amongst the three groups. The differences were confined to the Personal Inadequacies and the Relocation/Significant Separation subscales.
(15)	Klein (1998) and Cross sectional	U.S.A/Multiple sites in Northern California	Early (*n* = 11), middle (*n* = 22), and late (*n* = 24)	Early adolescence (12–14), middle adolescence (15–16), and late adolescence (17–21)	Age, race and ethnicity, marital status, employment status, education, participation in Adolescent Family Life Program	Shyness Scale and the Self-Esteem Scale, Perceived Maternal Expressiveness Instrument and the Perceived Paternal Expressiveness Instrument, PRQ-85-Part 11	Loneliness/UCLA Loneliness Scale	Situational loneliness, as measured by perceived maternal and paternal expressiveness, was expressed with greater significance than characterological loneliness. In the early, middle, and late age groups, there was a significant inverse relationship between social support and loneliness.
(16)	Yu et al. (2020) and Longitudinal	Obstetric clinics at a University Hospital	94	23.77 ± 4.42	Age. public insurance, race and ethnicity, relationship status, mental health, network characteristic, relationship type and closeness	Egocentric social network methods, EgoWeb software	Loneliness UCLA 3-Item Loneliness Scale	Completers and noncompleters did not differ on key characteristics. Social network density, but not social network size, predicted maternal loneliness in the first and third trimester.

*HRQoL, Health-related Quality of Life; LS, Life Satisfaction; SF-36, 36-Item Short Form Survey; SF-12, 12-Item Short Form Survey; CES-D, Center for Epidemiologic Studies Depression Scale; PROMIS, Patient-Reported Outcomes Measurement Information System; OHQ, The Oxford Happiness Questionnaire; SWLS, Satisfaction with Life Scale; SAS, Self-Rating Anxiety Scale; MSPSS, Multidimensional Scale of Perceived Social Support; MSC, Multiple Chemical Sensitivity*.

### Commonly Used SWB and SS Measurements

There are many measurements for SWB and SS across cultures. We have focused on the evaluative, hedonic, and eudemonic well-being, as well as on the perceived or received and structural or functional SS. The measurements used to assess these SWB and SS can be one-item or multi-item scale, qualitative or quantitative. Therefore, clarification is made in this section about the commonly used scales.

The satisfaction with life scale is the most commonly used index of SWB (Diener et al., 1985). This scale is used to assess overall LS by asking five questions, each of which involves a broad assessment of life. The personal wellbeing index (PWI; Cummins, 2014) takes a different approach. The PWI contains eight such domains which describe life as a whole. These are safety, health, the standard of living, achievement, future security, relationships, connection to community, and spirituality/religion. The 20-item positive and negative effect schedule (Watson et al., 1988) focused on positive effects such as excitement, enthusiasm, and inspiration. The Oxford happiness inventory scale (Hills and Argyle, 2002) is a scale that contains items on personality, optimism, control, self-esteem, and positive and negative effects. Hence, it is the most commonly used measurement of happiness. It correlates with almost any other well-being scale. For health-related QOL, the SF-36 scale (McHorney et al., 1993) is broadly used.

The multidimensional scale of perceived SS (Zimet et al., 1988) is a 12-item questionnaire using a 5-point Likert scale. It includes measures of perceived SS from family, friends, and significant others. The SS questionnaire (Sarason et al., 1983) assesses how people perceive SS. The quantity and types of connections inside a social network of an individual and the exchange of emotional, instrumental, or informational aid received from others can be assessed.

## Results

### Search Results

After removing duplicates, the original search generated 5,323 articles, leaving 4,948. After screening titles and abstracts, 86 eligible full texts were retrieved and screened. It was found that 34 studies were eligible according to our inclusion criteria. Four eligible abstracts could not be read in full as the authors did not respond to our full-text request or the manuscript had not been printed yet.

### Risk of Bias

The quality assessment tool of The National Heart, Lung, and Blood Institute for observational cohort and cross sectional studies statement was used to study the extent of bias in the studies (Table 4) (National Heart, 2018). This tool has been used in previous reviews (Ismaiel et al., 2019; López-Soto et al., 2019; Allevi et al., 2020; Kinshella et al., 2020; Larsen et al., 2020; Tarrant et al., 2020; Tinitali et al., 2021). The studies included in the present review were evaluated using this instrument. The reviewers (VM and JB) determined a total quality score for each article, and disagreements were successfully resolved during the discussion. About 61.7% of the studies use a cross sectional design. Due to the nature of the design, it increases the bias where several items cannot be applied. One strength of the selected articles is that they all used valid and reliable instruments for measuring an SWB outcome, as well as SS. The results varied from 4 to 13 out of a possible 14 points. Studies with a score of nine or above were deemed “good,” those with a score of from six to eight were deemed “fair,” and those with a score of five or less were deemed “poor” in terms of methodological quality.

**Table 4:** Summary of risk bias.

**References**	**Criteria**														**Quality rating**
	**(1)**	**(2)**	**(3)**	**(4)**	**(5)**	**(6)**	**(7)**	**(8)**	**(9)**	**(10)**	**(11)**	**(12)**	**(13)**	**(14)**	
Taşdemir et al. (2010)	YES	YES	YES	YES	NO	NA	NA	NA	YES	NA	YES	NA	NA	YES	Good
Robinson et al. (2018)	YES	YES	YES	YES	YES	NA	YES	YES	YES	YES	YES	NA	NA	YES	Good
Drescher et al. (2003)	YES	YES	NR	YES	NO	NA	NA	NA	NO	NA	YES	NA	NA	NO	Poor
Chang et al. (2014)	YES	YES	YES	YES	NO	NA	NA	YES	YES	YES	YES	NA	NA	YES	Good
Tesfa et al. (2020)	YES	YES	YES	YES	YES	NA	NA	NA	YES	NA	YES	NA	NA	YES	Good
Türk et al. (2017)	YES	YES	YES	YES	NO	NA	NA	NA	NO	NA	YES	NA	NA	YES	Fair
Ishfaq and Mushtaq (2019)	YES	YES	NR	YES	NO	NA	NA	NA	NO	NA	YES	NA	NA	NO	Poor
Malhotra et al. (2015)	YES	YES	NR	YES	NO	NA	NA	NA	YES	NA	YES	NA	NA	NO	Poor
Ndombe et al. (2011)	YES	YES	NO	YES	NO	NA	YES	YES	YES	YES	YES	NA	YES	NO	Good
Branecka-Wozniak et al. (2020)	YES	YES	NR	YES	NO	NA	NA	NA	YES	NA	YES	NA	NA	YES	Fair
Aasheim et al. (2014)	YES	YES	YES	YES	YES	NA	YES	YES	YES	YES	YES	NA	YES	YES	Good
Farooq et al. (2018)	YES	YES	NR	YES	NO	YES	NA	YES	YES	NA	YES	NA	NA	NO	Good
Lips (1985)	YES	YES	NR	YES	NO	NA	YES	YES	YES	YES	YES	NA	YES	YES	Good
Emmanuel et al. (2012)	YES	YES	YES	YES	YES	NA	YES	YES	YES	YES	YES	NA	YES	YES	Good
Ngai and Ngu (2013)	YES	YES	NR	YES	NO	NA	NA	NA	YES	NA	YES	NA	NA	YES	Fair
Gul et al. (2018)	YES	YES	NR	YES	YES	NA	NA	NA	YES	NA	YES	NA	NA	NO	Fair
Elsenbruch et al. (2007)	YES	YES	YES	YES	YES	YES	NA	YES	YES	NA	YES	NA	YES	YES	Good
Calou et al. (2018)	YES	YES	YES	YES	YES	NA	NA	NA	YES	NA	YES	NA	NA	YES	Good
MoghaddamHosseini et al. (2021)	YES	YES	YES	YES	YES	NA	NA	YES	YES	NO	YES	NA	NA	YES	Good
Macleod and Weaver (2003)	YES	YES	YES	YES	NO	NA	YES	YES	YES	YES	YES	NA	YES	YES	Good
Pakseresht et al. (2019)	YES	YES	NR	YES	YES	NA	NA	NA	YES	NA	YES	NA	NA	YES	Good
Stevenson et al. (1999)	YES	YES	NR	YES	NO	NA	NA	YES	YES	NA	YES	NA	NA	NO	Fair
Yu et al. (2020)	YES	YES	YES	YES	YES	NA	NA	NA	YES	NA	YES	NA	NA	YES	Good
Gebuza et al. (2014)	YES	YES	NR	YES	NO	NA	YES	YES	YES	YES	YES	NA	YES	NO	Good
Giblin et al. (1987)	YES	YES	YES	YES	NO	NA	NA	NA	YES	NA	YES	NA	NA	YES	Good
Nakamura et al. (2018)	YES	YES	NO	YES	NO	NA	YES	YES	YES	YES	YES	NA	NR	YES	Good
Rokach (2007)	YES	YES	NR	YES	NO	YES	NA	YES	YES	NA	YES	NA	NA	NO	Fair
Klein (1998)	YES	YES	NR	YES	NO	NA	NA	YES	YES	NA	YES	NA	NA	NO	Fair
Yu et al. (2020)	YES	YES	YES	YES	YES	NA	YES	YES	YES	YES	YES	NA	YES	YES	Good
Corno et al. (2018)	YES	YES	NR	NO	NO	YES	YES	YES	YES	YES	YES	NA	NA	NO	Fair
Hasanzadeh et al. (2020)	YES	YES	YES	YES	YES	YES	YES	YES	YES	YES	YES	NA	YES	YES	Good
Nazari et al. (2018)	YES	YES	NR	YES	YES	YES	YES	YES	YES	YES	YES	NA	YES	YES	Good
Cynthia Logsdon et al. (2005)	YES	YES	NR	YES	NO	NA	YES	YES	YES	YES	YES	NA	YES	NO	Good
Kazemi et al. (2017)	YES	YES	NR	YES	NO	NA	NO	NA	YES	NO	YES	NA	NA	YES	Fair

*NA, not applicable; NR, not reported*.

### Study Characteristics

The included studies covered a total of six domains of measurement, such as happiness, QoL, life satisfaction, positive and negative effects, well-being, and loneliness. These were grouped into the following: whether it measured the effect of pregnancy on the SWB of pregnant women, the effect of SS on the SWB of pregnant women, and whether an intervention or a qualitative approach was used. A total of 13 studies were included in the pregnancy itself (Lips, 1985; Drescher et al., 2003; Taşdemir et al., 2010; Ndombe et al., 2011; Aasheim et al., 2014; Chang et al., 2014; Malhotra et al., 2015; Türk et al., 2017; Farooq et al., 2018; Robinson et al., 2018; Ishfaq and Mushtaq, 2019; Branecka-Wozniak et al., 2020; Tesfa et al., 2020), 16 studies in SS (Giblin et al., 1987; Stevenson et al., 1999; Macleod and Weaver, 2003; Elsenbruch et al., 2007; Rokach, 2007; Emmanuel et al., 2012; Ngai and Ngu, 2013; Gebuza et al., 2014; Goodman et al., 2014; Calou et al., 2018; Gul et al., 2018; Nakamura et al., 2018; Pakseresht et al., 2019; Yu et al., 2020; MoghaddamHosseini et al., 2021), three interventions (Corno et al., 2018; Nazari et al., 2018; Hasanzadeh et al., 2020), and two qualitative studies (Cynthia Logsdon et al., 2005; Kazemi et al., 2017).

Descriptive analyses over the past 36 years (1985–2021) revealed an increasing research interest in the impact of pregnancy and SS on SWB with 24 studies of the 34 being published in the last decade. The studies were conducted in various countries across the globe, where the most common countries were Pakistan, Norway, China, and Iran. From each country, there were three (8.8%) studies. Three of the five intervention/qualitative studies were conducted in Iran. In regards to their design, studies varied little, with the vast majority being cross sectional studies (*n* = 21; 61.7%), followed by longitudinal studies (*n* = 8; 23.5%). In this review, three interventions and two qualitative assessments were included. The follow-up for the longitudinal studies was mostly done during and after pregnancy, commonly starting from their early pregnancy (*n* = 5; 14.7%). Follow-up time ranged greatly with an estimated time of 4 weeks (from the third trimester of pregnancy till after giving birth (Gebuza et al., 2014) to 3 years after birth (from gestational week 17 till 3 years after the birth; Aasheim et al., 2014).

Sample sizes varied from *n* = 6 (Corno et al., 2018) to *n* = 67,355 (Ndombe et al., 2011). The age of participants ranged from 16 (Drescher et al., 2003) to over 40 years (Tesfa et al., 2020). The most common age group in the studies included adolescent pregnancies (*n* = 8; 23.6%; Giblin et al., 1987; Stevenson et al., 1999; Drescher et al., 2003; Macleod and Weaver, 2003; Cynthia Logsdon et al., 2005; Taşdemir et al., 2010; Tesfa et al., 2020). Of the 20 studies which had mentioned education as a variable, in more than half of these studies (*n* = 14, 70%), the population was mostly educated with a high school diploma or higher. Among the population with primary education, there were illiterates or those who had not completed their school, and half of them (*n* = 6, 50%) were adolescent pregnancies, whereas the other half were adults. About 10 of the 17 studies had a population that either had a stable income or were employed. Among the studies which had assessed whether the mothers had spouses/partners or were single/divorced, almost all of them (*n* = 11, 91.7%) were married or had a stable relationship with their partner. Very few studies had assessed whether their current pregnancy was their first or if they had given birth before. Five out of the eight studies had a population where the majority was pregnant for the first time, whereas the other three studies had one or more children.

A great variety of SWB assessment tools was used. In total, 20 distinct SWB, happiness, and QoL questionnaires were used across studies with The Oxford Happiness Questionnaire (*n* = 6) and the 36-item short-form health survey (SF-36; *n* = 5) were the most recurring tools. SS was measured with 12 distinct questionnaires, the most common being the multidimensional scale of perceived SS (n = 4). Three studies used nondistinct questionnaires assessing sociodemographic characteristics, living arrangements, sources of financial support, adolescent partner of the female, family, friends during pregnancy, or use of health care services or anticipated assistance with child care. Two studies used in-depth semi structured personal interviews (Cynthia Logsdon et al., 2005; Nazari et al., 2018). One of the longitudinal studies reviewed the medical records of postpartum to ascertain pregnancy outcomes and subject information (Giblin et al., 1987). As a result, the changes in SWB and the effect of SS were assessed in different ways by using distinct standard questionnaires, by using medical records, or simply by inquiring about the QoL and SS of the participants.

### The Effect of Pregnancy Itself on SWB

The analysis is structured according to the different SWB metrics used in the papers. Starting from positive and negative effects, QoL, and happiness to LS.

Changes in SWB during pregnancy were measured by changes in positive and negative effects and emotions in three studies. According to one study, the mean degree of positive emotion during pregnancy at 12 weeks and 24–28 weeks of gestation scarcely varied (6.13 2.3 vs. 6.18 2.1), and there was no significant difference between the two time periods (Nakamura et al., 2018). Another study revealed that during pregnancy and the early postpartum period, a percentage of women reported an increase in negative emotion. Between the middle and the end of pregnancy, their depression ratings had risen considerably, which may reflect the increased physical stress of changing body form and weight as well as a sense that the pregnancy has gone on “forever” (Lips, 1985). In the third study, coping mechanisms, such as emotion-focused coping and problem-focused coping, proved to be ineffective predictors of positive and negative effects across pregnant participants (Farooq et al., 2018).

For both pregnant adolescents and adults, when measured across trimesters, QoL ratings were found to be greatest in the second trimester. Although, except the physical pain scale, all ratings were lower among teenagers when compared with adults (Taşdemir et al., 2010). Similarly, findings on the mental component summary (MCS) scores, vitality, and mental health had grown all across the three phases of pregnancy, with an increase in early to middle pregnancy and a reduction from middle to late pregnancy, according to another study (Chang et al., 2014). Two studies have found that QoL mean scores of pregnant women were significantly lower than women in the age-adjusted population norms. However, one report found no significant difference in the lower score (Drescher et al., 2003). Overall, the QoL of pregnant women is lower than the general population, but is at its highest during the second trimester, with a slight decline in the third trimester.

Happiness during pregnancy was found to be high with a mean of 74.01%. According to Malhotra et al. (2015), this height of happiness is characterized by sentiments of joy and self-fulfillment of women. The experience of being pregnant appears to have made them regard life as highly fulfilling and full of joy. Age-wise, it was reported that younger women have more subjective happiness (4.19 ± 1.67) than older pregnant women (3.11 ± 1.24). The results show significant differences between the groups of younger and elder pregnant women (Ishfaq and Mushtaq, 2019). Malhotra et al. (2015) and Türk et al. (2017) reported similar results in their research. The odds of being unhappy (poor degree of happiness) among pregnant women were higher in their third trimesters (AOR; 1.89, 95% CI; 1.19, 3.01) than among women in their first trimesters. Unintended pregnancy and intimate relationship violence were two other indicators of a lower degree of happiness (Tesfa et al., 2020). According to Malhotra et al. (2015), pregnant women living in joint families were happier than those living in nuclear households, with 75.8 and 71.2% reportedly experiencing contentment [contentment (75.8%) and ??? (71.2%)]. There is a missing term to compare with, respectively.

Life satisfaction during pregnancy was high among the mothers, with scores ranging from 1 to 10 (8 on average; Branecka-Wozniak et al., 2020). Controversial results were reported about LS level changes during pregnancy. While one study found that satisfaction increased during pregnancy and infancy, peaking at 6 months postpartum (Ndombe et al., 2011), another found that the mean LS scores were similar regardless of age at the first timepoints (gestational weeks 17 and 30, and 6 months), but were significantly lower 3 years later (Aasheim et al., 2014). The third research found that as pregnancy develops, the LS of pregnant women (*p* < 0.05) declines (Branecka-Wozniak et al., 2020). Age was also found to be another component affecting LS, where during gestational weeks 17, 30, and 6 months, LS increased from the age of 25–28 years eventually, dropping around 40 years of age and over (Aasheim et al., 2014).

### The Effect of SS and Pregnancy on SWB

The results have been structured according to the effect SS had on pregnancy, as well as on the different SWB metrics used. It focused on the effect of SS on pregnancy itself, on QoL, happiness, LS, and at last on loneliness.

The effect of social support on SWB among pregnant women was measured by two studies. One of them found that positive emotion at 12 weeks of gestation to have a positive correlation with health-related QOL and good family functioning. This differed significantly between the positive emotion-decrease and the positive emotion-increase group (Nakamura et al., 2018). Positive interactions with the teenage father throughout pregnancy were linked to getting sufficient prenatal care and good attitudes about the pregnancy from relatives and friends, as well as the overall happiness of the adolescent with living circumstances (Giblin et al., 1987).

Several studies found that SS had a significant positive correlation with HRQoL (*p* < 0.01; Elsenbruch et al., 2007; Emmanuel et al., 2012; Ngai and Ngu, 2013; Gul et al., 2018; Nakamura et al., 2018). One study reported that other than HRQOL, SS had a significant positive correlation to physical functioning (*p* < 0.01), emotional well-being (*p* < 0.05), and energy/fatigue (*p* < 0.01) (Gul et al., 2018). In another study, mothers with partners reported higher SS ratings, whereas those without spouses reported lower scores (Emmanuel et al., 2012). Calou et al. (2018) summarized that the relationship with the partner and the family influenced the health-related QoL of pregnant women. Persons with whom the woman lives/anxiety for the birth of the baby (*p* = 0.029), parity/relationship with the family (*p* = 0.005), occupation/self-esteem (*p* = 0.000), marital status/relationship with the partner (*p* = 0.029), and partner support/relationship with the partner (*p* = 0.018) were predictors that positivity influenced the QoL (Calou et al., 2018). A better feeling of family coherence was found to be essential in another study, as it was linked to a higher degree of SS, a higher QoL (mental health component), reduced stress, and fewer depressive symptoms during pregnancy (Ngai and Ngu, 2013). In a study measuring the QoL during pregnancy, the eight dimensions of HRQoL revealed means for the relevant age group that was lower than the population norms, which after birth, despite the improvements, remained below the general population norm (Emmanuel et al., 2012). Similarly, pregnant women with high SS scored higher compared with the published German reference values, whereas, the results of the poor SS group were substantially lower than the norm (both *p* < 0.001; Elsenbruch et al., 2007).

The effect of SS on happiness was assessed in two studies, one study in which it was found that happiness had a significant relationship with the support of the husband/support of the parents (*p* = 0.001), marital satisfaction (*p* = 0.001), and education of the husband (*p* = 0.003), as well as age (*p* = 0.001), occupation (*p* = 0.029), and monthly income (*p* = 0.001) (Pakseresht et al., 2019). It was observed that roughly 80% of the respondents in the older group and roughly 60% of respondents in the younger group identify their partners as a source of SS. At 37 weeks, both age groups expressed equal levels of satisfaction with SS (Macleod and Weaver, 2003). At 20 weeks gestation, participants included 3.45 ± 2.10 individuals in their SS networks, that is, three to four people, and at 37 weeks, the number was 3.45 ± 2.10, that is, three to four people. The author did, however, find that the “scale of the social network” had no impact on “satisfaction with social support,” “attitudes,” or “happiness” metrics. They explained that SS from their family was plentiful, with over 80% of the respondents claiming to have received it from either their moms or both parents during pregnancy. In addition, support from friends dwindled by the 37th-week interview, and SS of the healthcare workers was noticeably lacking (Macleod and Weaver, 2003). Overall, SS from their husband, followed by family and friends, has a substantial influence on the happiness of pregnant women. The quality of the happiness of a mother with their assistance was more important than the quantity.

Life satisfaction was positively associated with SS (*r* = 0.576, *P* < 0.01; Yu et al., 2020). Another study found that among the forms of SS received during pregnancy, emotional support was high among the group with a “high degree of support,” which accounted for 85% of the observations. This implies that the women in the study were surrounded by caring, protecting, and empathic significant others, according to the author. As for “instrumental support received,” it consisted of 82%, and “information support received” consisted of 60%. Finally, emotional support was shown to have the strongest connection between assistance received and LS (Gebuza et al., 2014). In another study among adolescent pregnant women and SS from their parents, 44 (40.4%) had a bidirectional exchange of support, 19 (17.4%) were providers, 13 (11.9%) receivers, and 33 (30.3%) low supporters. Bidirectional trended toward higher life satisfaction, less anxiousness, and less depression than others (Stevenson et al., 1999). Hence, the LS of pregnant women can be higher if the support is given as well as it is received. There were no significant changes in any of the well-being measures between teenagers dating the father of their child and dating an individual other than the father of the child, or those without a partner (Stevenson et al., 1999).

Loneliness is another important measure of SS among pregnant women. When comparing loneliness among pregnant, new mothers, and the general population, scores of pregnant women indicated that they felt lonelier than the general population. Defying Rokach's (Rokach, 2007) expectations of Rokach, there was no significant difference in loneliness score between pregnant and new mothers on any subscales. Yu et al. (2020) discovered that the size of the social network of an individual had no significant impact on maternal loneliness. Social network density, on the other hand, was shown to be negatively associated with the loneliness of the mothers. In other words, people who had more linked social networks reported experiencing less loneliness. There was a substantial negative association between SS and loneliness among early adolescence (12–14), middle adolescence (15–16), and late adolescence (17–21) age groups. The study also assessed the characterological and situational variables concerning loneliness and had found them to be significant in the total sample. In particular, perceived maternal expressiveness and SS had a significant positive relationship between the situational variables of loneliness (Klein, 1998).

### Intervention, Qualitative Assessment of SWB, and SS

Three interventions were conducted to improve happiness among pregnant women (Table 5). Fordyce happiness training, focused on self-efficacy, was the subject of research of Nazari et al. (2018), whereas Hasanzadeh et al. (2020) applied attachment training to their study. There was a substantial distinction between the pretest and posttest mean happiness levels of the two groups in both the investigations (Nazari et al., 2018; Hasanzadeh et al., 2020). The study revealed that without the training, there was a substantial reduction in the happiness median score and an increase in perceived stress ratings at the conclusion of the trial when compared with preintervention data. Furthermore, an inverse correlation between stress and happiness was observed (Nazari et al., 2018). A 5-week, self-applied, internet program named “positive pregnancy” was undertaken in another study. For each unit, including a brief psychoeducation unit centered on a positive psychology facet and exercise, the findings revealed that, on average, the mental well-being of a women and LS rose from pre- to postintervention (Corno et al., 2018).

**Table 5:** The intervention and qualitative assessments of SS, SWB among pregnant women.

**Author/Year**	**Country**	**Study design**	**Intervention/Interview**	**Sample size**	**Mean age**	**Dependent variable**	**Outcome measurement**	**Result of intervention/interview**
Corno et al. (2018)	Not listed	Prospective study	“Positive Pregnancy” is a 5-week, self-applied, web-based program designed with each module includes a brief psychoeducation unit focused on a positive psychology dimension and a positive psychology exercise.	6	N/A	Well-being	The Warwick–Edinburgh Mental Well-Being Scale, PHQ-92, The Pregnancy-Related Anxiety Scale, The Satisfaction with Life Scale, The Multidimensional Scale of Perceived Social Support	On average, women's mental well-being levels increased from pre- to postintervention. Women's levels of satisfaction with life increased from pre- to postintervention
Hasanzadeh et al. (2020)	Iran	Prospective study	The intervention group received attachment training through six 90-min sessions, while the control group underwent the hospital's routine care. The two groups were required to fill out the study questionnaires once more after 4 weeks after the intervention	84	29.49 ± 4.28	Happiness	Oxford Happiness Questionnaire, Cranley's Maternal-Fetal Attachment Scale	There was a significant difference between the two groups' pretest and posttest mean scores of happiness. The difference between the two groups was statistically significant
Nazari et al. (2018)	Iran	Prospective study	Fordyce Happiness Cognitive-Behavioral training based on self-efficacy was done in the intervention group and there was no intervention for the control group.	100 (50, 50)	Intervention group: 28.80 ± 4.89, Control group: 26.98 ± 5.16	Happiness	Oxford Happiness Inventory, Perceived Stress Questionnaire	The results of the Wilcoxon Test indicated a significant difference in the intervention group's median scores of happiness and perceived stress before and after the intervention
Cynthia Logsdon et al. (2005)	U.S.A	Qualitative	The rationale for meeting twice was to capture information about social support needs as the adolescent was adjusting to pregnancy and again as the time of the birth drew closer.	30	13–14 years old (*n* = 6), 15–16 years old (*n* = 19), 17–18 years old (*n* = 5)	Social support	(a) What is the experience of receiving social support like? and (b) How does the need for social support, and the ability to negotiate social support, vary by age?	SS for pregnant adolescents varied depending on their family, socioeconomic status, threats to safety, and relationship with the baby's father. The younger adolescents felt that their parents recognized that they still needed to act like a child. Older pregnant adolescents had more realistic expectations, such assist them to apply for college and/or to obtain a job.
Kazemi et al. (2017)	Iran	Qualitative	Data were collected through face-to-face in-depth semistructured interviews. During interviews, searching questions were asked in order to further clarify participants' views.	16	29.69 ± 5.03	QoL	“What crosses your mind when you hear the word QOL?” “How has your QOL been since you fell pregnant?” “What negative effects has pregnancy had on your QOL?” “Which negative effects of pregnancy on your QOL have worried you”? “When is a pregnant woman's QOL considered good?”	All participants referred to their husbands' greater attention and support from them during pregnancy as major factors contributing to their stress reduction. Pregnant women's mothers played significant roles in reducing their stress by providing them with emotional support. Some of them also expressed their satisfaction with healthcare services by their physicians and midwives.

The study concluded that SS for pregnant teenagers differed based on their family, financial position, safety risks, and connection with the father of the baby, while applying a qualitative method. The mothers of the pregnant teenagers were more constant sources of assistance than their fathers. Even though they were pregnant, the younger ones (13–14 years old) among the adolescents felt that their parents realized that they still needed to behave similar to a child. The connection of an adolescent with the father of the baby was characterized by a significantly high level of ambivalence. The older pregnant teenagers (17–18 years old), on the other hand, had more practical SS needs. They desired assistance in applying for college and/or finding employment (Cynthia Logsdon et al., 2005). In another study among participants with a mean age of 29.69 ± 5.03, participants referred to greater attention and support of their husbands and an adequate understanding of pregnancy as a major factor contributing to their stress reduction and QoL improvement. Apart from spouses, other family members, notably the mothers of pregnant women, played an important role in providing emotional support with, for example, soothing words (Kazemi et al., 2017). Some of them also reported contentment with the medical attention provided by their doctors and midwives, mentioning their attentiveness and professionalism as factors in their serenity (Kazemi et al., 2017).

## Discussion

The relationship between SWB and SS in pregnant women is not well defined. Based on the content researched for this review, this is the first systematic review to assess SS and its effect on SWB in healthy pregnant women. In this regard, we summarized key findings and the research landscape.

The studies employed a variety of SWB and SS outcome measures. Due to the infeasibility of utilizing a meta-analysis, this review focused on summarizing the similarities and differences in the outcomes and comparing these studies within the methodology. The results show some general patterns, despite the variation in measures.

The principal finding of the present systematic review is that pregnancy by itself has a positive association with happiness (Malhotra et al., 2015; Türk et al., 2017; Ishfaq and Mushtaq, 2019); however, the other SWB assessments had diverse results. SS during pregnancy has a positive association with all measurements of SWB (Macleod and Weaver, 2003; Elsenbruch et al., 2007; Emmanuel et al., 2012; Ngai and Ngu, 2013; Gebuza et al., 2014; Gul et al., 2018; Nakamura et al., 2018; Pakseresht et al., 2019; Yu et al., 2020).

### Pregnancy on SWB

The different measurements of SWB show different results during pregnancy. Happiness and LS were found to be high with a mean of 74.01% or a score of 8 out of 10. These results are consistent with the SWB homeostasis theory. It is proposed that SWB bears some resemblance to body temperature homeostasis and that it is actively regulated and maintained (Cummins and Nistico, 2002). The theory mentions that it has several characteristics which help the general sense of well-being to be held remarkably positive at a nonspecific, abstract level. One of its characteristics is that each person has a stable positive mood state level that is set genetically to a specific “set-point” (Davern et al., 2007). It lies high amongst the dissatisfied–satisfied continuum, specifically in the “satisfied” section (Cummins et al., 2002). As a result, when participants are asked “How satisfied are you with your life as a whole?”, on a scale of 0 to 100, the average set-point is 75 among western nations (Cummins, 1995, 1998). These responses are quite similar to the results discovered by the researchers of this review. One question used in the satisfaction with life scale of the included studies is “I am satisfied with my life,” which is the same as the one in the theory above. Hence, the LS assessment of SWB is consistent in both theory and practice. Another feature of LS is that it is stable. Even if exceptionally good or terrible, events create changes in the near term, and homeostasis will generally restore global contentment with life to its prior level over time. These results show different changes throughout pregnancy. In several of these studies, LS remained stable while in others, it either increased or decreased. According to the theory, these changes are short term and might have returned to the previous level of LS if the studies had done a follow-up again after birth. We have found that during pregnancy, the QoL and positive emotion had decreased and that the mean scores of QoL among pregnant women were lower than women in the age-adjusted population norms. In these studies, the results showed the overall QoL, involving both the mental and physical components, whereas, the study which separately focused on the different components of the questionnaire had discovered an increase in MCS scores throughout the three stages of pregnancy, in contrast with a decline in physical component summary scores. A number of studies support the statement of similar results (Tendais et al., 2011; Dalfrà et al., 2012; Do et al., 2017; Bai and Raat, 2018), as almost all of them had found a decrease in physical HRQoL with moderately increased MCS scores. The physical changes in the body throughout the pregnancy have negatively affected the mental component and the happiness level of the population. As stated in this study the odds of being unhappy were almost two times higher among women in their third trimesters than those in the first trimesters. It is consistent with the results of an SWB review, which stated that “conditions that cause physical, psychological, economic, and social suffering, which impact SWB” (Das et al., 2020).

From the interventions conducted among pregnant women, most focused on the measurements of depression, stress, and anxiety (Barber et al., 2013; Kim et al., 2014; Bright et al., 2019) or on postpartum depression (Danaher et al., 2013; O'Mahen et al., 2014; Pugh et al., 2016). To our knowledge, other than the three studies in this review according to our inclusion criteria, only a few sought to improve SWB (Abdos et al., 2018). These studies were conducted to improve SWB, and happiness has shown that Fordyce happiness cognitive-behavioral training (Nazari et al., 2018), attachment training (Hasanzadeh et al., 2020), and “positive pregnancy” (Corno et al., 2018) improved the median score and decreased perceived stress scores. A positive psychology aspect (i.e., awareness and self-acceptance, relishing, connectivity, supports, positivity, and life happiness) and a positive psychology practice were the emphases of the programs (Corno et al., 2018). The findings of this review suggest that focused effort on a positive psychology dimension and positive psychology exercises had positive mental health outcomes among pregnant women.

### SS of Pregnant Women on SWB

This review found that SS had a more evident positive association with SWB than pregnancy did, even if the variables were isolated. SS was often measured using both objective (e.g., the size of the “social circle” of an individual) and subjective (e.g., quality or satisfaction with social contacts). We paid equal attention to both aspects.

A review that summarized the theoretical tenets of SWB has stated one of the seven determinants/correlates of SWB. These are the following: SS, fundamental demographics, financial position, health and functioning, character, spirituality and culture, and location and infrastructure (Das et al., 2020). The ability of SS from family, community, and friends and acquaintances to buffer the impact of possible stresses on SWB has been almost widely recognized in research and literature reviews (Henderson, 1977; Sarason, 1990; Cramm et al., 2010; Khan and Husain, 2010). Other publications have indicated that the quality of relationships (Pinquart and Sörensen, 2000; Sandstrom and Dunn, 2014) influences SWB. Similarly, in this review, we found that the more the number of social contacts and the satisfaction the pregnant women experienced from the relationship, the more was a salient impact on happiness (Macleod and Weaver, 2003). Most of the studies in the review stated that most women ranked their partner as the main source of support, followed by their family members. Support from mothers was extremely essential for teens, and “emotional support” had the strongest link between LS and support received.

Social support is one of the defenses against negative environmental interactions that could prevent the SWB homeostasis to fail and set the SWB set-point below its normal range (Cummins et al., 2010). Cummins et al. (2010) stated that a “relationship with another human being that involves mutual sharing of intimacies and support” is the most important buffer of balance. Das et al. (2020) stated that SS may also be more critical to SWB for individuals with health difficulties compared with the general norms. Hence, SS during pregnancy has a very strong impact on SWB. Although there are reviews about SS during pregnancy (Gjerdingen et al., 1991; Orr, 2004; Hodnett et al., 2010; East et al., 2019), to our knowledge, only one publication was found assessing both QoL and SS among pregnant women (Lagadec et al., 2018). This study concluded that SS was one of the main factors associated with QoL. It also shows that SS during pregnancy may reduce the likelihood of depression, which might enhance SWB and pregnancy outcomes (Lagadec et al., 2018). In this review, we have found that pregnant women with more SS positively correlated with adequate levels of prenatal care, physical functioning, emotional well-being, health-related QoL, life satisfaction, and happiness.

The current analysis also reveals a strong link between loneliness and its adverse association with SS (Klein, 1998). The research of SS and loneliness has many published reviews, of which, the results are consistent with ours (Hawkley and Cacioppo, 2010; Wang et al., 2018). However, different from the expectation of the author of decreased loneliness after birth, the results showed no significant difference in loneliness scores between pregnant and new mothers on any subscales (Rokach, 2007). It means that there was no difference found in the source of loneliness among the pregnant women and the new mothers with an additional member to the family.

The sort of SS received differed based on their family, social level, safety risks, and connection with the father of the baby, according to the qualitative results of pregnant women. Among the younger pregnant women, they had stated their parents to be a major emotional support, while the older parents wanted SS for their future aspirations, such as obtaining a job.

The main contribution from this systematic review is that SS influences SWB whereas pregnancy by itself had varying results. Based on the evidence in this study, further research and interventions can be done to gather the necessary data and clarify the exact role of pregnancy and SS on SWB. Among the publications, much is about depression, anxiety, and stress. Therefore, other rigorous and systematic study designs, interventions (including evaluation of positive psychology), SWB, the measurement effectiveness of health services (public or private) for pregnancy care, and the quality-adjusted life years should be considered. These are likely to be the key factors in explaining possible differences between SS and pregnancy, and how this affects SWB in developed and developing societies.

### Limitations

Concerning this review, several limitations were identified. First, several SWB measures included in the study seemed to be the most logical. However, we recognized that more SWB terms might have been incorporated as well. Nevertheless, considering the complexity of the topic of these studies, we propose that these initial measurements as specific keywords can map out content about SWB. Moreover, there is the second limitation in this regard. While the inclusion of 34 studies evaluated seven different aspects of SWB, SS measurements and their relationship to each other are a notable strength of this research in mapping out the landscape of this topic. Due to the vast scope, the narrative synthesis of data does not reflect the full breadth and complexity of the pregnancy/SWB/SS connection. Rather, it attempts to highlight the emerging patterns and trends across research on a more basic level. The third limitation, a sole author examined the entire text to see if it was eligible. The inclusion of arguable studies was discussed among the research group if they fit the inclusion criteria until consensus was reached. Fourth, in the included studies, we did not conduct a quality appraisal. As a result, while scoping the research landscape, all studies were allotted the same relevance. Fifth, we attempted to reduce the possibility of missing any relevant articles that were published in a different language or in publications that were not featured in the databases by cross referencing three separate databases. We may have overlooked potentially relevant gray literature (e.g., dissertations, government documents, and white papers) because we only examined peer-reviewed journal articles. Sixth, research focused on specific positive psychology (e.g., happiness, well-being, LS, etc.) but not utilizing the sought terms may have been missed by scanning for phrases like SWB or happiness. We attempted to reduce this specific danger once again by cross referencing. Finally, among the variables of the pregnant women, this review found that the majority of studies included pregnant women, who are educated, married, or had a partner who was either employed or had a stable income. Women with other socioeconomic characteristics are underrepresented, leading to a slight overestimation of SWL in the review.

## Conclusion

This scoping review presents an overview of the existing state of research on the influence of pregnancy and SS on SWB, as well as a synopsis of significant results. SS had a significant effect on SWB among pregnant women. While the association of pregnancy itself with SWB is more variable, generally LS, happiness, and the mental component of QoL are high during pregnancy but positive emotion and physical components of QoL decreased through the pregnancy. In this context, during pregnancy SWB tends to change for both better and for worse, while SS during this time can act as a buffer to the negative changes. Despite certain emerging trends, the general profile of research on this subject remains diverse and fragmented due to the lack of conceptual and terminological clarity, as well as disparate study methodologies and reporting. As a result, it is difficult to combine and evaluate data from different investigations. Last, we believe that these findings will contribute to the advancement of research techniques in the critically important domains of SWB and SS.

## Resource Identification Initiative

(PubMed Central, RRID:SCR_004166)

(Google Scholar, RRID:SCR_008878)

## Data Availability Statement

The original contributions presented in the study are included in the article/supplementary material, further inquiries can be directed to the corresponding author.

## Author Contributions

BB contributed to the conception, design of the study, investigation, and writing of the original as well as editing and reviewing of the manuscript. EB-E and MB worked equally on the data curation, investigation, and reviewing. HC acted as the supervisor and reviewed as well as edited the manuscript. All authors contributed to manuscript revision, read, and approved the submitted version.

## Conflict of Interest

The authors declare that the research was conducted in the absence of any commercial or financial relationships that could be construed as a potential conflict of interest.

## Publisher's Note

All claims expressed in this article are solely those of the authors and do not necessarily represent those of their affiliated organizations, or those of the publisher, the editors and the reviewers. Any product that may be evaluated in this article, or claim that may be made by its manufacturer, is not guaranteed or endorsed by the publisher.
